# Identification of the intersegmental plane via electromagnetic navigation for anatomical segmentectomy

**DOI:** 10.1111/1759-7714.15030

**Published:** 2023-07-16

**Authors:** Tong Qiu, Wenjie Jiao, Yandong Zhao, Yunpeng Xuan

**Affiliations:** ^1^ Department of Thoracic Surgery The Affiliated Hospital of Qingdao University Qingdao China

**Keywords:** anatomical segmentectomy, electromagnetic navigation, intersegmental plane, lung cancer

## Abstract

Accurate identification of the physiological intersegmental plane is crucial for successful anatomical segmentectomy. Current techniques, such as the inflation‐deflation method, may result in uncertain cutting lines, leading to unsuitable resection extents. Here, we demonstrated the successful use of electromagnetic navigation with methylene blue dye‐marking to preoperatively and precisely identify the physiological intersegmental plane in two patients with small‐sized peripheral non‐small cell lung cancer (NSCLC). This novel technique offers the potential for precise cutting lines that align closely with the physiological intersegmental plane, thus improving the accuracy and efficacy of anatomical segmentectomy for these selected NSCLC patients.

## Graphical abstract

This is a novel technique for preoperative and precise identification of the intersegmental plane by using electromagnetic navigation with methylene blue dye‐marking, offering precise cutting lines that align closely with the physiological intersegmental plane, thus improving the accuracy and efficacy of anatomical segmentectomy for selected NSCLC patients.

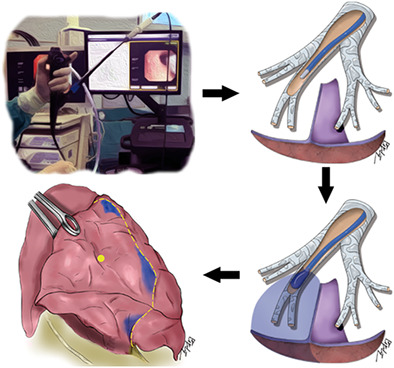

## INTRODUCTION

For the management of small‐sized peripheral non‐small cell lung cancer (NSCLC), identification of the physiological intersegmental plane (ISP) is a crucial step in the execution of anatomical segmentectomy. While inflation‐deflation^1^ and systemic indocyanine green (ICG) injection^2^ are widely employed by thoracic surgeons, a pathophysiological ISP may occasionally arise in patients afflicted by severe emphysema, heavy smoking history, or due to prior lung parenchyma injury from surgical manipulation.^3^ In complement to these existing techniques, we present two cases in which successful anatomical segmentectomy was achieved using preoperative identification of the physiological ISP through electromagnetic navigation bronchoscopy (ENB). The ethics committee has given the ethics approval for this manuscript (no. QYFYKY 2018‐10‐11‐2).

## CASE REPORT

**FIGURE 1:**
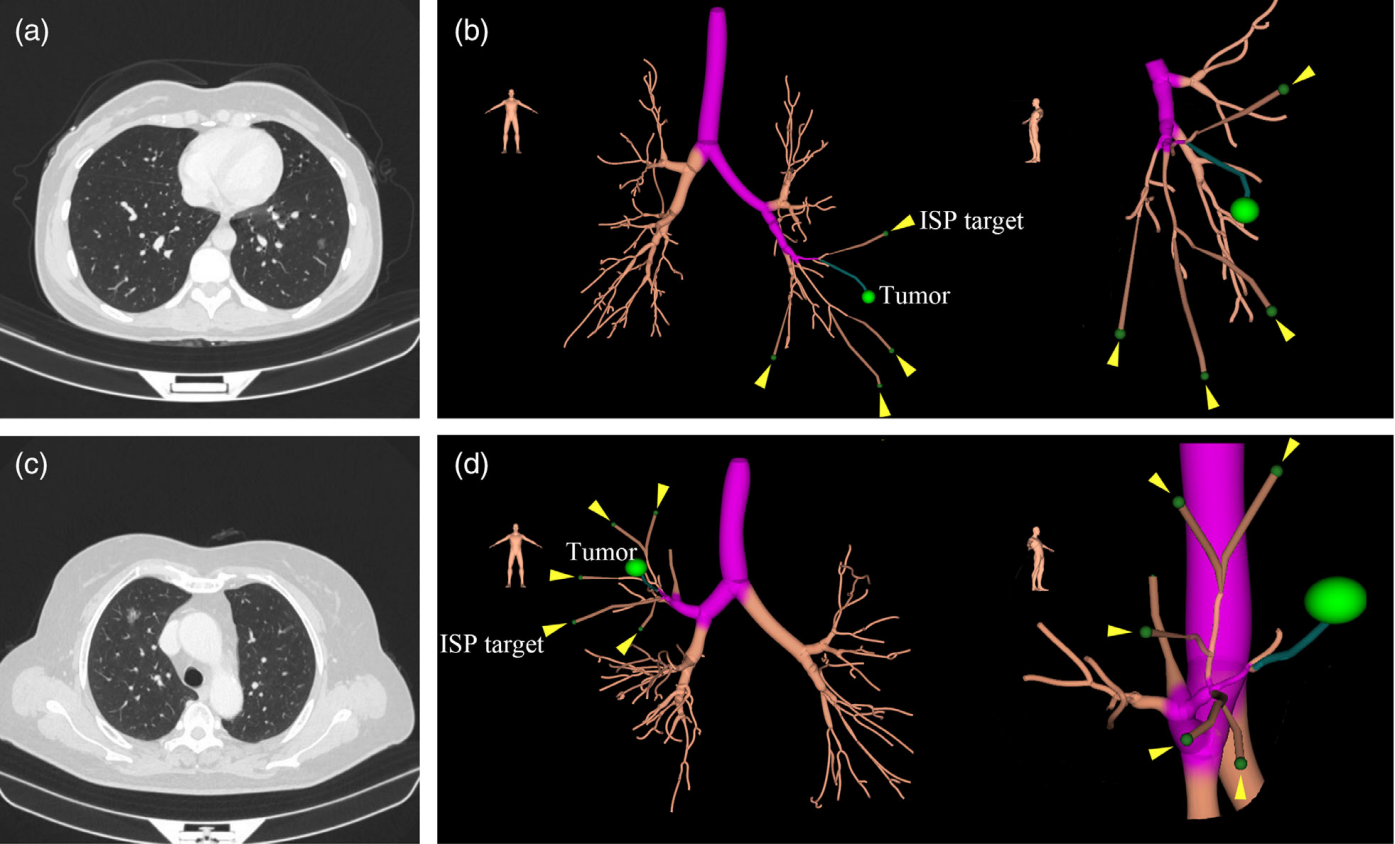
The ENB planning process. (a) CT findings in case 1. (b) The ISP targets* in case 1 between the LS8 and the adjoining segments of LS6/LS9/LS10. (c) CT findings in case 2. (d) The ISP targets in case 2 between the RS3 and the adjoining segments of RS1/RS2. * The ISP targets (yellow arrow) were set at the terminal of the bronchus (approximately 1 cm under the visceral pleura), belonging to the sub‐subsegments of the target segment. CT, computed tomography; ENB, electromagnetic navigation bronchoscopy; ISP, intersegmental plane.

Case 1 was a 42‐year‐old female, who was admitted with a peripheral 10 mm pure ground‐glass nodule in the left S8 segment (LS8) (Figure 1a). For preoperative ENB‐guided identification of the ISP, the DICOM file of the chest computed tomography (CT) with 0.625 mm of thickness was imported to the superDimension version 7.0 (superDimension Inc., USA). During the planning process, the lesion was marked on the virtual map of the bronchial tree, and the ISP targets were set at the terminal of the bronchus which were approximately 1 cm under the visceral pleura, belonging to the sub‐subsegments of LS8 and adjoining segments of LS6, LS9, and LS10 (Figure 1b).

Case 2 was a 70‐year‐old male with a 15 mm ground‐glass nodule in the right S3 segment (RS3) (Figure 1c). Using the same method, the ISP targets at the terminal of the bronchus were set, which belonged to the sub‐subsegments of RS3 and adjoining segments of RS1 and RS2 (Figure 1d).

**FIGURE 2:**
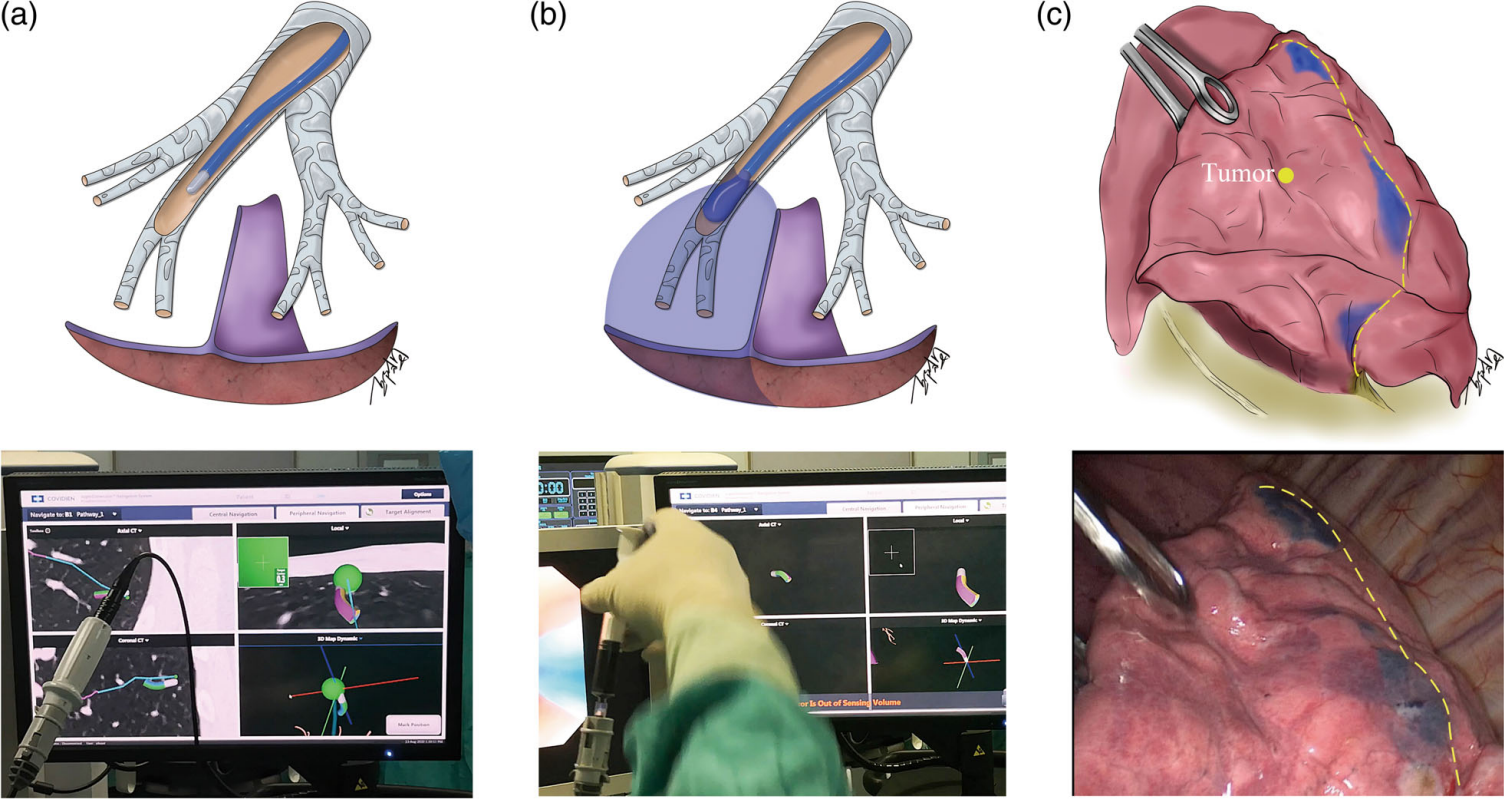
The procedure of preoperative ENB‐guided identification of the ISP. (a) The ENB locatable guide reaches the ISP target. (b) The process of dye‐marking. (c) Intraoperative scene of the interrupted dye‐marked areas connected with the virtual line, which served as the physiological ISP and cutting lines. ENB, electromagnetic navigation bronchoscopy; ISP, intersegmental plane.

The ENB procedure was performed before the surgery in the operation room on the operating day. Following the ENB registration process, the locatable guide (LG) with an extended working channel (EWC) was delivered through bronchoscopy. Under electromagnetic navigation, LG/EWC was locked to hold its position once it reached each ISP target. Then LG was retracted and the EWC was kept in place. After injecting 0.5 mL methylene blue into the EWC, the LG was immediately inserted into the EWC. The LG/EWC remained in place for a few seconds to enable diffusion of the methylene blue into the subpleural parenchyma. Upon dye‐marking all ISP targets, the ENB procedure was concluded (Figure 2a,b).

After undergoing the ENB procedure, the patient was placed in the right decubitus position with single lung ventilation for a two‐port approach thoracoscopic segmentectomy. During the operation, the interrupted dye marks on the visceral pleura between the target segment and the adjoining segments were detected (LS8 vs. LS6/LS9/LS10 in case 1, and RS3 vs, RS1/RS2 in case 2). With the lesion as the reference point, a virtual line connecting the lateral margins of the stained areas was created, which served as the physiological ISP between the target segment and the adjacent segments (Figure 2c). We proceeded with the anatomical segmentectomy of LS8 for case 1 and RS3 for case 2 without utilizing the inflation‐deflation method. The ISP was dissected using endo‐staplers along the previously established virtual line following the anatomical dissection of the segmental bronchus, artery, and vein. The frozen section examination subsequently confirmed the diagnosis of adenocarcinoma with lepidic predominant pattern in case 1 and microinvasive adenocarcinoma in case 2. The ENB procedure took approximately 16 min in case 1 and 13 min in case 2, while the operation duration was 67 min in case 1 and 59 min in case 2.

## DISCUSSION

The identification of the physiological ISP represents a significant technical challenge that needs to be resolved before segmentectomy can become the standard procedure for small‐sized peripheral NSCLC.^4^, ^5^ Currently, the most commonly used method of identifying the ISP relies on differential air distribution between the targeted and residual segments.^1^, ^6^ In recent years, infrared thoracoscopy following injection of ICG has also been reported.^7^, ^8^ However, these methods are in essence post‐processing procedures, following the surgical manipulation of the lung parenchyma and single‐lung ventilation, which might lead to the risk of creating pathophysiological ISP in the injured lung.^9^, ^10^

The ENB procedure is comprised of three fundamental techniques: three‐dimensional reconstruction, virtual localization, and electromagnetic navigation. We previously described the vectorial localization of peripheral pulmonary lesions using electromagnetic navigation, demonstrating that the LG/EWC could precisely reach the sub‐pleura.^11^ Owing to its demonstrated accuracy and efficiency, ENB with methylene blue dye‐marking has emerged as a viable alternative for preoperative pulmonary nodule localization.^12^ Furthermore, the methylene blue solution could diffuse into pulmonary lobules, forming a polygonal blue area with sharp margins in the sub‐pleura. By harnessing the advantageous properties of ENB and dye diffusivity, it is possible to preoperatively and precisely mark the outermost lobules of the target segment in physiological status. With these dye‐marked landmarks, the physiological ISP could be visualized once the thoracotomy is made, without any surgical and ventilating‐related injury to the lung. In addition, the ENB technique also avoids intraoperative waiting time for inflation‐deflation or injection of ICG, thus reducing surgery‐related lung injury. Therefore, this novel technique of identifying ISP via electromagnetic navigation might provide accurate cutting lines closer to the physiological ISP, potentially improving the accuracy and efficacy of anatomical segmentectomy for selected NSCLC patients.

## AUTHOR CONTRIBUTIONS

All authors had full access to the data in the study and take responsibility for the integrity of the data and the accuracy of the data analysis. Study concept and design: T.Q. and W.J.J. Acquisition of data: T.Q., Y.D.Z, Y.P.X. Analysis and interpretation of the data: T.Q. and W.J.J. Drafting of the manuscript: T.Q. and W.J.J. Obtained funding: T.Q. Study supervision: W.J.J.

## CONFLICT OF INTEREST STATEMENT

The authors confirm that there is no conflict of interest.

## Supporting information


**Video S1.** Identification of intersegmental plane via electromagnetic navigation for anatomical segmentectomy in case 1.
